# Efficacy of Novel Noncontrast Cardiac Magnetic Resonance Methods in Indicating Fibrosis in Hypertrophic Cardiomyopathy

**DOI:** 10.1155/2021/9931136

**Published:** 2021-05-24

**Authors:** Maedeh Sharifian, Nahid Rezaeian, Sanaz Asadian, Ali Mohammadzadeh, Ali Nahardani, Kianosh Kasani, Yaser Toloueitabar, Ali Mohammad Farahmand, Leila Hosseini

**Affiliations:** ^1^Rajaie Cardiovascular Medical and Research Center, Iran University of Medical Sciences, Tehran, Iran; ^2^Tehran University of Medical Sciences, Tehran, Iran

## Abstract

**Objective:**

In hypertrophic cardiomyopathy (HCM), myocardial fibrosis is routinely shown by late gadolinium enhancement (LGE) in cardiac magnetic resonance (CMR) imaging. We evaluated the efficacy of 2 novel contrast-free CMR methods, namely, diffusion-weighted imaging (DWI) and feature-tracking (FT) method, in detecting myocardial fibrosis.

**Methods:**

This cross-sectional study was conducted on 26 patients with HCM. Visual and quantitative comparisons were made between DWI and LGE images. Regional longitudinal, circumferential, and radial strains were compared between LGE-positive and LGE-negative segments. Moreover, global strains were compared between LGE-positive and LGE-negative patients as well as between patients with mild and marked LGE.

**Results:**

All 3 strains showed significant differences between LGE-positive and LGE-negative segments (*P* < 0.001). The regional longitudinal and circumferential strain parameters showed significant associations with LGE (*P* < 0.001), while regional circumferential strain was the only independent predictor of LGE in logistic regression models (OR: 1.140, 95% CI: 1.073 to 1.207, *P* < 0.001). A comparison of global strains between patients with LGE percentages of below 15% and above 15% demonstrated that global circumferential strain was the only parameter to show impairment in the group with marked myocardial fibrosis, with borderline significance (*P*=0.09). A review of 212 segments demonstrated a qualitative visual agreement between DWI and LGE in 193 segments (91%). The mean apparent diffusion coefficient was comparable between LGE-positive and LGE-negative segments (*P*=0.51).

**Conclusions:**

FT-CMR, especially regional circumferential strain, can reliably show fibrosis-containing segments in HCM. Further, DWI can function as an efficient qualitative method for the estimation of the fibrosis extent in HCM.

## 1. Introduction

Hypertrophic cardiomyopathy (HCM) is the most common genetic cardiac disorder with autosomal dominant inheritance and heterogeneous patterns of penetration and expression [1, 2]. The condition is characterized by left ventricular (LV) hypertrophy with no other probable causative etiologies. The clinical presentation varies from the absence of symptoms to exertional dyspnea, chest pain, syncope, and sudden cardiac death. In many cases, sudden cardiac death due to ventricular tachyarrhythmia is the first presentation [3]. Myocardial fibrosis occurs in more than half of this patient population [4] and is an underlying cause of ventricular tachyarrhythmia [5]. Cardiac magnetic resonance (CMR) imaging is able to detect the presence and extent of fibrosis manifested as areas of late gadolinium enhancement (LGE). Areas of LGE are compatible with fibrosis on histopathology [6]. The explanatory mechanism for LGE is the widening of extracellular spaces in fibrotic areas, which leads to the temporary distribution of gadolinium and relative hyperenhancement by comparison with adjacent healthy tissues [7]. The 2020 guidelines of the American Heart Association/American College of Cardiology (AHA/ACC) for the diagnosis and management of HCM suggest LGE as a useful risk modifier for sudden cardiac death among patients in whom the risk is deemed borderline based on conventional risk stratification [8]. Nonetheless, the need for contrast injection limits the utilization of LGE in patients with contrast allergy and renal insufficiency, which explains why recent years have witnessed an increase in research on the usefulness of contrast-free techniques such as diffusion-weighted imaging (DWI), native T1, and extracellular volume (ECV) mapping.

The results of a study on infarcted swine hearts indicated comparable accuracy between DWI, LGE, and histology in detecting and delineating densely scarred areas, border zone areas, and healthy tissues [9]. A few small-scale studies have shown that not only can DWI depict fibrosis in HCM hearts but also its results exhibit a good correlation with LGE findings [10–12]. There have also been investigations suggesting the superiority of DWI over LGE owing to such probable advantages as the capability to show both diffuse scarring in the early stages of HCM [10, 12] and fibrotic areas that are not well visualized by LGE [12]. Additionally, the relatively novel technique of feature-tracking cardiac magnetic resonance (FT-CMR) has proven its efficaciousness in some cardiac disorders by assisting in diagnosis, risk stratification, and prognostication [13–18]. Since FT-CMR is a contrast-free method and is feasible through a postprocessing analysis of routine cine images, it can act as a practical, available, and cost-beneficial method in the diagnosis of areas with myocardial fibrosis.

In the present study, we studied 26 patients with HCM to evaluate the efficacy of the 2 novel techniques of DWI and FT-CMR in demonstrating areas with myocardial fibrosis.

## 2. Methods

### 2.1. Study Population

The current investigation enrolled 30 consecutive patients with HCM who were referred to our center for CMR between July 2019 and December 2019. All these patients provided informed written consent for participation in the project. The study protocol was approved by the Medical Research Ethics Board of Iran University of Medical Sciences.

The exclusion criteria were a history of previous cardiac surgeries, including myectomy; contraindications for CMR such as severe contrast allergy, severe renal failure (glomerular filtration rate <45 mL/min/1.73 m^2^), and the presence of cardiac devices such as internal cardioverter defibrillators and pacemakers; coronary artery disease; systemic hypertension; severe valvular disease; and systemic diseases affecting the myocardium such as amyloidosis.

### 2.2. Diagnostic Criteria of CMR

The present study recruited adult patients with a maximal end-diastolic LV wall thickness of 15 mm or greater in the absence of other etiologies for LV hypertrophy or 13 mm or greater in the presence of a positive family history or genetic test for HCM [8].

### 2.3. Cardiac Imaging Protocol

All the patients underwent a comprehensive study in keeping with a prespecific routine CMR imaging protocol using a 1.5 T MRI machine (MAGNETOM Avanto, Siemens Healthcare, Erlangen, Germany) and a vendor-supplied body surface coil. The imaging protocol consisted of the acquisition of 2-, 3-, and 4-chamber views, short-axis cine (functional) images, and LGE images 10 minutes after contrast injection. Also, breath-hold low *b*-value myocardial DWI spin-echo echo-planar imaging (EPI) sequences were performed on all the patients with an interactive ECG-gating regime to collect all signals at the freezing point of myocardial contraction (repetition time = 90 ms, echo time = 54 ms, flip angle = 90°, pixel bandwidth = 2220 KHz, resolution = 1.45 × 1.45 mm^2^, slice thickness = 10 mm, average = 1, *b* = 0 s/mm^2^, and *b* = 100/150 s/mm^2^).

### 2.4. Image Analysis

#### 2.4.1. LGE Sequence Interpretation

The presence of LGE in each patient and each different segment was detected by an expert with more than 5 years of experience in cardiac imaging. With the aid of CVI42 software, the LGE percentage was calculated for each patient as the sum of hyperenhanced regions with +5SD signal intensity above the normal remote myocardium divided by the total LV myocardial mass, expressed as the percentage of the enhanced myocardial mass. The patients were classified into 2 groups: marked fibrosis (fibrosis percentage ≥15%) and mild fibrosis (fibrosis percentage <15%).

#### 2.4.2. FT-CMR Analysis

**Figure 1:**
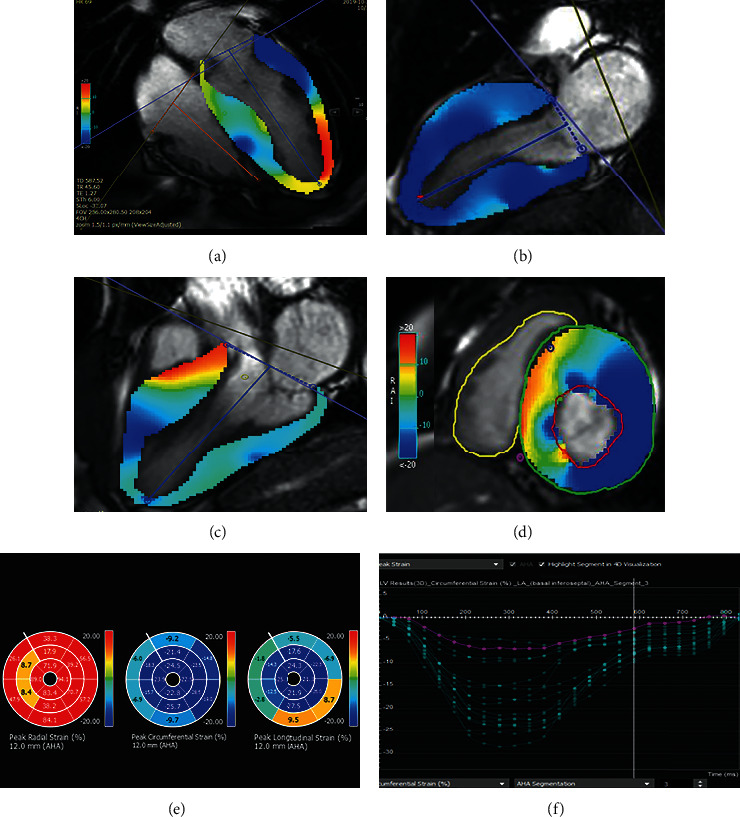
CMR feature-tracking technique for determining myocardial strain. (a) Four-chamber, (b) two-chamber, (c) three-chamber, and (d) short-axis images depict left ventricle endocardial (red) and epicardial (green) borders. (e) Bull's eye maps and (f) strain curves according to AHA myocardial segmentation.

Circle CVI's imaging platform, cvi42 (Calgary, Canada), was used to measure 3D longitudinal, circumferential, and radial strain parameters from cine images. First, brightness was adjusted to reach the optimal discrimination of the endocardium and blood pool. Next, endocardial and epicardial borders were defined on 3 long-axis views (2-, 3-, and 4- chamber views) and short-axis views (Figure 1). The next step saw the propagation of the contours before the calculation of regional strain values for the 16 AHA segments as well as the calculation of global strain values with the aid of the software. For ease, the absolute amounts of strain values were used.

#### 2.4.3. DWI and Apparent Diffusion Coefficient (ADC) Analysis

**Figure 2:**
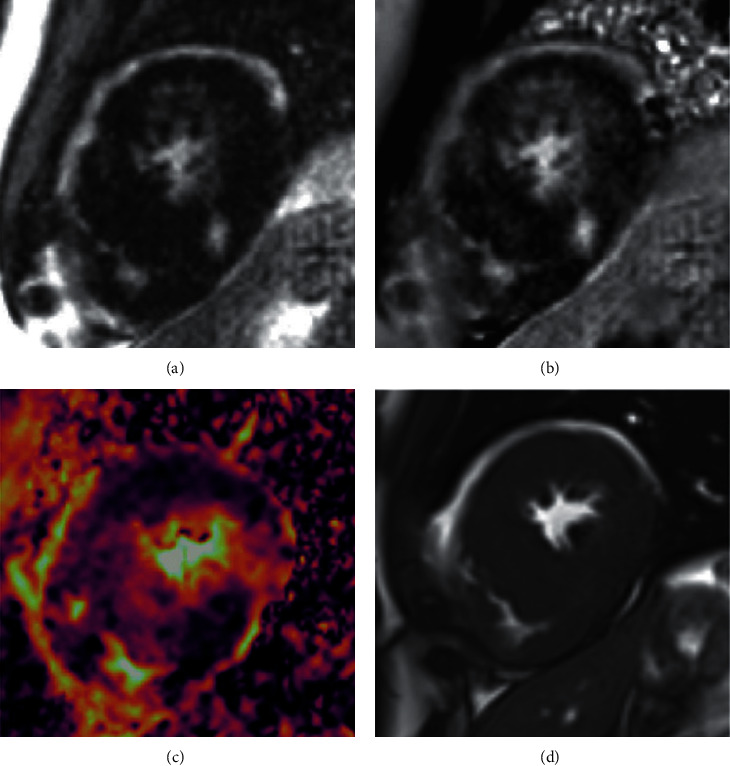
(a) Late gadolinium enhancement sequence (magnitude image), (b) late gadolinium enhancement sequence (phase image), (c) ADC map, and (d) true FISP image in a known case of hypertrophic cardiomyopathy. ADC: apparent diffusion coefficient; FISP: fast imaging with steady-state free precession.

DWI images were taken from the middle and apex of the heart for the evaluation of 6 middle and 4 apical AHA cardiac segments. The DWI images were evaluated via both qualitative and quantitative methods. For the qualitative evaluation, a radiologist and a cardiologist, who were both experienced in the field and blinded to the patients' history and other CMR sequences, independently observed the DWI images and classified the segments as fibrosis-positive and fibrosis-negative (intraobserver reproducibility: 0.89–0.92). In the case of disagreement between the 2 interpreters, the final decision was made by the senior radiologist in the ward. These visual assay findings were thereafter compared with those of segmental LGE. In the next step, ADC maps were calculated from the DWI dataset using MATLAB software, and the mean ADC value for each AHA segment was extracted (Figure 2).

### 2.5. Statistical Analysis

SPSS software, version 22.00, was used for the statistical analyses. Continuous variables with normal distributions were described as the mean ± the standard deviation (SD), while categorical variables were expressed as frequencies and percentages. Intergroup comparisons between LGE-positive and LGE-negative cases and between LGE < 15% and ≥15% were performed in terms of the quantitative variables by using an independent-samples *t*-test. The predictive power of regional strain parameters for regional LGE was tested using logistic regression models. A 2-tailed *P* value of less than 0.05 was considered statistically significant.

## 3. Results

### 3.1. Patients and Baseline Characteristics

The current study recruited 30 patients, of whom 4 were excluded due to severe motion artifacts. Twenty-six patients (female: 30.8%) at a mean (±SD) age of 46.5 (±14 y) were studied. The systolic anterior motion of mitral valve leaflets was seen in 13 patients (50%). Mild-to-moderate valvular abnormalities were seen in 11 patients (42.3%), with mitral regurgitation being the most common abnormality (*n* = 9), followed by pulmonary insufficiency (*n* = 4). LGE was present in 20 patients (76.9%: LGE-positive cases) and 52 out of 212 available segments (24.5%).  Table 1 displays the baseline demographic and cardiac MRI characteristics of the total study population and subgroup comparison based on the LGE percentage of greater or less than 15%. The patients with LGE percentage of 15% or greater had significantly lower LV ejection fraction (47 ± 13% vs. 58 ± 8%, *P*=0.024) and cardiac output (4 ± 0.8 L/m vs. 5.3 ± 1.4 L/m, *P*=0.027), as well as significantly higher LV end-systolic volume index (46 ± 23 ml/m^2^ vs. 29 ± 13 ml/m^2^, *P*=0.032) and maximal wall thickness (24 ± 6.2 mm vs. 17 ± 4.1 mm, *P*=0.003). The LV mass index and LV end-diastolic volume index did not show a significant difference between the two groups (*P* > 0.1 for both).

### 3.2. FT-CMR Analysis

The comparison of global strain values between LGE-positive and LGE-negative patients demonstrated no significant difference in global longitudinal strain (GLS), global radial strain (GRS), and global circumferential strain (GCS) (GLS = 11 ± 3.5 vs. 12.3 ± 4.5, GCS = 14.3 ± 3.0 vs. 15.2 ± 4.4, and GRS = 36.9 ± 14.0 vs. 31.3 ± 14.3, respectively; *P* > 0.1 for all). According to their LGE percentage, the patients were divided into 2 groups of LGE of less than 15% and LGE of 15% or greater so that global strain values could be compared between the groups. The intergroup analysis showed no significant difference in the absolute global strain values (GLS = 10.60 ± 3.60 in LGE ≥ 15% vs. 12.42 ± 3.72 in LGE < 15%, *P* > 0.1; GRS = 36.52 ± 18.40 in LGE ≥ 15% vs. 35.20 ± 12.26 in LGE < 15%, *P* > 0.1; and GCS = 12.67 ± 2.57 in LGE ≥ 15% vs. 15.33 ± 3.40 in LGE < 15%, *P*=0.09). Thereafter, a comparison of segmental longitudinal, circumferential, and radial strain values revealed significant differences concerning all 3 strain parameters between LGE-positive and LGE-negative segments (longitudinal strain = 10.5 ± 5.1 vs. 15.5 ± 6.6, circumferential strain = 11.8 ± 5.7 vs. 16.8 ± 6.3, and radial strain = 24.8 ± 26.6 vs. 32.2 ± 28.1, respectively; *P* < 0.001 for all). As is shown in Table 2, the logistic regression models revealed that regional longitudinal and circumferential strain values had significant associations with regional LGE (*P* < 0.001 for both), and circumferential strain was the only independent predictor of segmental LGE (OR: 1.140, 95% CI: 1.073 to 1.207, *P* < 0.001).

### 3.3. DWI and ADC Analysis

In 12 patients, the DWI images of cardiac apical segments were excluded due to severe motion artifacts. In the total of 212/260 reviewed segments, a visual agreement between DWI and LGE images was reported in 193 segments (91%). In the remaining 19 segments, 5 LGE-positive segments were classified as fibrosis-negative by DWI images (false negative) and 14 LGE-negative segments were classified as fibrosis-positive by DWI images (false positive). In 11 out of the 19 discordant segments (57%), the timing of the cardiac cycle was dissimilar between LGE and DWI images. The mean ADC was 0.64 ± 0.25 for LGE-positive and 0.61 ± 0.23 for LGE-negative segments. The difference between the ADC values of LGE-positive and LGE-negative segments failed to constitute statistical significance (*P*=0.51).

## 4. Discussion

The main findings of our investigation are as follows:

1. All regional strain values showed significant deterioration in fibrosis-containing segments
2. Among the 3 regional strain values, circumferential strain was an independent predictor of fibrosis
3. In patients with notable amounts of fibrosis, GCS showed relative impairment
4. The qualitative assessment of DWI images revealed an excellent agreement with LGE sequences
5. The quantitative assessment of ADC showed no difference between segments with or without LGE

Contractile dysfunction in HCM and its relationship with tissue characteristics have been the focus of some previous studies. Using speckle-tracking echocardiography, Popovic et al [19] demonstrated that regional and global longitudinal strain parameters had decreased absolute values in LGE-containing segments and patients, respectively. In their study, regional circumferential and radial strain values were comparable between LGE-positive and LGE-negative segments. Conversely, in a more recent study on 45 patients with HCM, Wu et al. [11] reported impairment in all 3 regional strain parameters in LGE-positive segments and segments with increased ECV values. Similarly, in a study on pediatric HCM, Bogarapu et al [20] demonstrated that all strains were significantly decreased in LGE-positive groups and introduced a cutoff point of −12.8% for GLS to differentiate between LGE-positive and LGE-negative patients. Scintillatingly, Swoboda et al [21] reported that segmental hypertrophy, LGE, and abnormal native T1 and ECV values were all associated with impairment in circumferential and radial strains. However, they showed that after correction for regional wall thickness, circumferential and radial strain values were significantly associated with native T1, but not with LGE and ECV. Therefore, they proposed that contractile abnormality was probably affected more by cellular changes and to a lesser extent by extracellular expansion.

In our study, global strains exhibited no significant difference between LGE-positive and LGE-negative cases. The regional strains, however, showed significant impairment in LGE-positive segments, and segmental circumferential strain proved to be an independent predictor of LGE. This might be due to the fact that, in HCM, myocardial fibrosis is typically midwall [4, 22, 23], and among the 3 strain axes, it is circumferential strain that is mainly generated by the circumferential fibers of the midwall myocardium [24]. Consequently, circumferential strain is diminished by the fibrous degeneration of these fibers. The mean maximal wall thickness of the patients in our study was low by comparison with that reported by some previous studies [20]. Given that the myocardial thickness is a major determinant of disease severity in HCM, it is probable that our study group consisted of individuals with milder disease. Thus, it could be postulated that, in the course of HCM, circumferential strain is the first strain to show impairment.

A few previous studies have utilized DWI to detect fibrosis in HCM hearts [10–12]. Our results demonstrated that the visual identification of fibrotic regions on b100 DWI images was consistent with LGE images in 91% of the reviewed segments, and more than half of the observed discordance was attributable to mismatched cardiac phases. Since no more than 2 segments were discordant in DWI with LGE images in each patient, the total visual estimation of fibrosis was comparable between LGE and DWI images. This is in line with the results of Nguyen et al [10], who reported a high visual agreement between ADC, ECV, and LGE images, not least for the detection of patch-like fibrosis. Nevertheless, in our study, ADC values did not show a significant difference between LGE-positive and LGE-negative segments. The broad SD of the ADC values in both normal and fibrotic regions indicates that low *b*-value DWI is not an efficient quantitative technique for the evaluation of myocardial changes. This finding does not chime in with that reported by a few investigations that demonstrated significantly increased ADC measures in fibrosis-positive segments defined by either LGE or ECV images [10–12]. One possible explanation for this discrepancy could be the fact that the low *b*-value diffusion also contains data regarding the perfusion characteristics of the myocardium [25, 26]. As long as resting-state dynamic contrast-enhanced myocardial perfusion images are close to normal, it is logical to observe no ADC value changes in the study group. On the other hand, according to previous studies, abnormal rest perfusion and reduction in capillary microcirculation are seen in a large percentage of patients with HCM and are mostly associated with areas of fibrosis shown in LGE images [27]. We, therefore, hypothesized that the reduction in the microcirculation in these areas might compensate for the increase in movements in the widened ECV and result in no significant change in the ADC values of these areas.

### 4.1. Limitations

The current study has some notable limitations, first and foremost among which is our small sample size, possibly explaining the indeterminate significance of some findings. In our view, similar studies with larger samples should be considered. Second, DWI sequences were based on segmented spin-echo EPI, which added to the susceptibility of the lung tissue over the LV lateral wall and caused distortion in some images. Third, EPI-based sequences usually have a low signal-to-noise ratio, adding to the complexity of image and data analysis. Thus, we recommend the evaluation of balanced steady-state free precession for DWI in larger studies on HCM.

## 5. Conclusions

FT-CMR can reliably show fibrosis-containing segments in HCM. Circumferential strain is probably the first strain parameter to exhibit impairment in LGE-positive patients. This is of paramount importance since this novel postprocessing technique can serve as an available, easy, and fast method to show myocardial fibrosis in clinical practice and obviate the need for any additional CMR sequences or contrast injections. DWI can function as an efficient qualitative method for the estimation of the fibrosis extent in HCM, whereas low *b*-value DWI is not a reliable method to quantify the scar extent. Further large-scale multicentric studies are warranted to reliably propose these 2 novel noncontrast CMR methods for fibrosis prediction in patients suffering from HCM.

## Figures and Tables

**Table 1:** Baseline demographic and cardiac MRI characteristics of the study population.

Variables	All subjects (*n* = 26)	LGE < 15% (*n* = 18)	LGE ≥ 15% (*n* = 8)	*P* value
Age (y)	46.6 (±14)	47.1 (±13)y	45.2 (±19)	0.776
Gender (female)	8/26 (30.8%)	3/18 (16%)	5/8 (62%)	0.060
Body surface area (m^2^)	1.91 (±0.21)	1.9 (±0.16)	1.7 (±0.23)	**0.006**
Positive family history	17/26 (68%)	14/18 (77%)	3/8 (37%)	0.078
Heart rate	64 (±8)	65 (±9)	63 (±6)	0.522
LVEF (%)	54 (±11)	58 (±8)	47 (±13)	**0.024**
LVESVI (mL/m^2^)	34.7 (±18.3)	29 (±13)	46 (±23)	**0.032**
LVEDVI (mL/m^2^)	74.7 (±20.8)	70 (±19)	84 (±22)	0.121
LV mass index (g/m^2^)	84.1 (±39.1)	78 (±35)	96 (±46)	0.312
Cardiac output (L/m)	4.9 (±1.4)	5.3 (±1.4)	4.0 (±0.8)	**0.027**
Maximal wall thickness (mm)	19.5 (±5.7)	17 (±4.1)	24 (±6.2)	**0.003**

Values are mean (±SD) or *n* (%). LVEF: left ventricular ejection fraction; LVESVI: left ventricular end-systolic volume index; LVEDVI: left ventricular end-diastolic volume index; LV: left ventricle.

**Table 2:** The results of logistic regression analysis on the predictive role of regional strain values for regional fibrosis.

Variable	OR (95% CI)	*P* value
Regional longitudinal strain	1.121 (1.052–1.183)	**<0.001**
Regional circumferential strain	1.125 (1.052–1.183)	**<0.001**
Regional radial strain	1.005 (0.993–1.016)	0.386

## Data Availability

The datasets generated during the current research are available from the corresponding author upon reasonable request.
